# Hepatitis E Virus Genotype 3 Strains in Domestic Pigs, Cameroon

**DOI:** 10.3201/eid1904.121634

**Published:** 2013-04

**Authors:** Vanessa Salete de Paula, Matthias Wiele, Afegenwi H. Mbunkah, Achukwi M. Daniel, Manchang T. Kingsley, Jonas Schmidt-Chanasit

**Affiliations:** Fundação Oswaldo Cruz, Rio de Janeiro, Brazil (V. Salete de Paula);; Bernhard Nocht Institute for Tropical Medicine, Hamburg, Germany (V. Salete de Paula, M. Wiele, J. Schmidt-Chanasit);; Centre Pasteur, Yaoundé, Cameroon (A.H. Mbunkah);; Institute of Agricultural Research for Development, Ngaoundere, Cameroon (A.M. Daniel, M.T. Kingsley)

**Keywords:** hepatitis E virus, HEV, viruses, subtype, genotype, pigs, Cameroon

**To the Editor:** Hepatitis E virus (HEV) is a positive-stranded, non-enveloped RNA virus of the family *Hepeviridae* that is considered to be the main causative agent of enterically transmitted acute hepatitis (*1*). HEV is classified into 4 genotypes (*1*). HEV genotypes 1 and 2 cause large waterborne epidemics of acute hepatitis in developing countries, especially in Africa and Asia (*1*). In contrast, HEV genotypes 3 and 4 are increasingly identified as causative agents of acute viral hepatitis in industrialized countries (*1*). Genotypes 1 and 2 are found only in humans, whereas genotypes 3 and 4 are associated with food-borne zoonotic transmission from domestic pigs, wild boar, and deer (*1*).

In addition to these 4 genotypes, HEV-related viruses were detected in avian, rodent, and bat hosts, which formed novel genera within the family *Hepeviridae* (*2*). In Africa, HEV genotype 1 and 2 strains have been identified during HEV epidemics (*3**–**5*). An HEV genotype 3 strain was detected in 1 of 40 fecal samples from domestic pigs in Kinshasa, Democratic Republic of the Congo, and it was suggested that this strain was imported from Belgium to the Democratic Republic of the Congo by animal trade (*6*). Therefore, we investigated whether HEV strains of genotype 3 or 4 are circulating among domestic pigs in Cameroon.

During February–March 2012, a total of 345 liver samples were collected from domestic pigs (age range 6 months–3 years) in abattoirs in Douala and Yaoundé, Cameroon, and in slaughter slaps (areas) in Bamenda, Cameroon. Pigs were mainly of the local breed. In addition, pigs originating from extensive cross-breeding (local X landrace and local X Duroc) were sampled. Liver samples were collected during post-mortem inspection.

Viral RNA was extracted from liver samples by using the RTP DNA/RNA Virus Mini Kit II (STRATEC Molecular, Berlin, Germany) according to the manufacturer’s instructions. Extracted RNA was analyzed for HEV RNA by using 2 nested reverse transcription PCRs (RT-PCRs) specific for open reading frame 1 (OFR 1) and ORF 2 of HEV (*7**,**8*). Nested RT-PCRs and direct sequencing of amplicons were performed as described (*9*). RNA of HEV strain Hamburg-HB (GenBank accession no. JN986840) was used as a positive control for nested RT-PCRs.

HEV RNA was detected in 2 samples from female pigs in Yaoundé (2/139) and 1 sample from a male pig in Bamenda (1/39). All 167 samples from Douala were negative for HEV RNA. The sample from Bamenda showed a positive result for the nested RT-PCR specific for HEV ORF 1. Genetic distances calculated with partial nucleotide sequences of ORF 1 (280 nt) and ORF 2 (373 nt) between strain Yaounde56 and the most closely related HEV genotype 3 strains from Japan (JSWINE150-Aom04R; GenBank accession no. AB221520) and Mongolia (swMN06-A1354; GenBank accession no. AB290105) were 90% and 91%, respectively.

At the amino acid level, the partial RNA-dependent RNA polymerase sequence (ORF 1) and the partial capsid protein sequence (ORF 2) of strain Yaounde56 were identical to those of HEV genotype 3 strains HEV/Gt3/HSD40/2009 (GenBank accession no. AFO71833) from Germany and swJ12–1 (GenBank accession no. BAC66273) from Japan. Thus, all mutations were silent.

In agreement with distance analysis, phylogenetic reconstruction using partial nucleotide sequences of ORF 2 (278 nt) showed a close relationship of strains Yaounde56 and Yaounde94 with HEV genotype 3 strains (Figure). However, the HEV strains from Cameroon do not cluster with the classified HEV genotype 3 subtype reference strains (*10*) in the phylogenetic tree (Figure). These strains cluster within a clade of subtype undefined strains and are most closely related to strain swMN06-A1354 from Mongolia (Figure).

**Figure:**
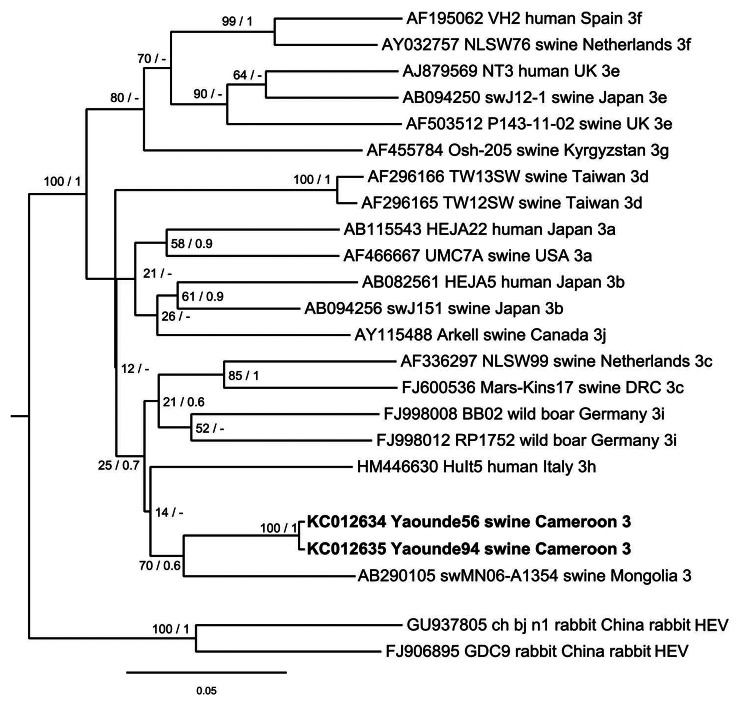
Phylogenetic analysis of hepatitis E virus (HEV) strains, Cameroon. The Bayesian phylogenetic tree was constructed by using partial nucleotide sequence of open reading frame 2 (278 nt) of HEV. For each sequence used, the GenBank accession number, strain designation, source of isolation, country of isolation, and HEV subtype are shown. Multiple nucleotide sequence alignment was analyzed by using the Markov Chain Monte Carlo method implemented in the program MrBayes version 3.0 (http://mrbayes.sourceforge.net/) and applying the general time-reversible substitution model. Posterior probabilities are shown at the nodes of the tree to the right of the slash if >0.5. Bootstrap values calculated from 10,000 replicates are indicated at the nodes of the tree to the left of the slash. Alignment was analyzed by using the neighbor-joining method and resulted in same tree topology (not shown). Newly described HEV sequences are shown in **boldface**. Scale bar indicates evolutionary distance. UK, United Kingdom; USA, United States; DRC, Democratic Republic of Congo.

Because the pig production cycle is shorter than that for cattle, pig production is a major economic activity in Cameroon. Most pigs in Cameroon are local raised, and extensive cross-breeding is used. The infection rate of pigs with HEV genotype 3 strains from Cameroon is lower than that of pigs from Europe. Thus, HEV genotype 3 seems to have an extensive distribution that includes Africa. Future studies should investigate how HEV genotype 3 strains contribute to sporadic HEV cases in Cameroon.
